# Are cattle dangerous to walkers? A scoping review

**DOI:** 10.1136/injuryprev-2015-041784

**Published:** 2016-01-12

**Authors:** Angharad P Fraser-Williams, K Marie McIntyre, Carri Westgarth

**Affiliations:** 1 Faculty of Health and Life Sciences, School of Veterinary Science, University of Liverpool, Leahurst Campus, Cheshire, UK; 2 Faculty of Health and Life Sciences, Department of Epidemiology and Population Health, Institute of Infection and Global Health, University of Liverpool, Leahurst Campus, Cheshire, UK; 3 Institute for Risk and Uncertainty, University of Liverpool

## Abstract

Anecdotal evidence suggests that people coming into contact with cattle while participating in outdoor pursuits can sustain severe, even fatal injuries. This has negative implications for farmers, cattle and the public. This study outlines findings from a review of published literature, UK media reports and internet guidelines currently available to the UK public for walking near cattle. A total of 54 cattle attacks were reported in the UK media from 1 January 1993 to 31 May 2013; approximately one-quarter resulted in fatality and two-thirds involved dogs. Walking with dogs among cows, particularly with calves present, was a problematic context. Twenty pieces of commonly occurring advice were found within various guidelines. However, there are no definitive approved guidelines, no published studies describing the prevalence of cattle attacks on members of the public and no system in place to document them. Attacks by cattle are underinvestigated and further work should assess their public health impact.

## Introduction

Annually, there are 3.6 billion tourist visits to the UK countryside; 18% of visitors being walkers.^1^ ^2^ In England in 2011–2012, dog walking was the second most frequent countryside activity, accounting for 51.0% of visits.^3^ As promotion of physical activity via dog walking is a public health strategy, evaluation of potential risks to public health from these activities is important.^4^

There are approximately 300 000 UK farms^5^and 9.7 million cattle.^6^ Public rights of way cross farmland including livestock grazing, so that while walking people come into contact with livestock, particularly cattle, and may have limited knowledge of how to behave around them.

Risk assessments and mitigation measures are increasingly implemented to manage risk, but intensive attempts to govern human/cattle interactions would have negative implications for farmers, cattle and the public. Likely risk-management practices include removal of herds from publically accessed fields during peak seasons or restricting public access, and euthanasia of cattle that have attacked walkers. These could affect animal welfare via husbandry and farming practices, and financially impact farmers. Further, they could reduce public motivation for countryside outdoor activities. Thus, it is important to fully evaluate the scope of this public health problem.

The aim of this project was to assess available information about negative interactions between the public and cattle, to identify risk factors for cattle attacks, and highlight the availability and usefulness of guidance on walking among livestock. The study outlines the findings of: (1) a literature review of published research; (2) a review of media reports; (3) an internet search of guidelines for countryside walking.

## Methods

### Search-term development

Search terms for sets of searches were developed by iteratively trialling popular related phrases found in the published literature, media reports and using internet searches, until the most commonly occurring search terms were identified. Using this method, ‘cow’ for example was identified as being more commonly used than ‘cattle’, and sufficient to return all findings also related to ‘cattle’. The searches were conducted by a single author.

### Published literature

**Figure 1:**
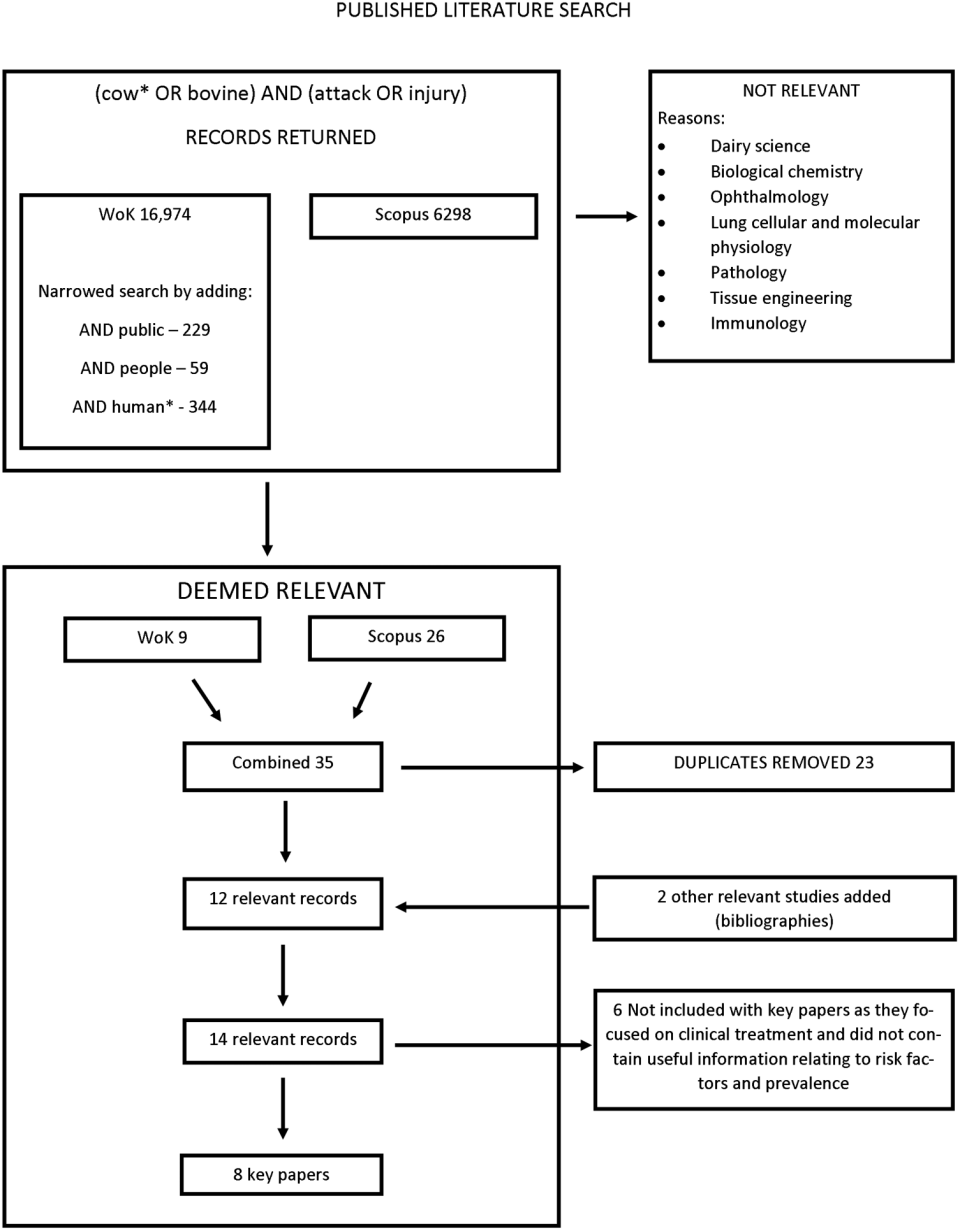
A schematic diagram of the search phrases used with the SCOPUS and Web of Knowledge (WoK) bibliometric databases, including the number of records returned during a search of published literature relating to outcomes and risk factors of cattle attacks on people undertaking outdoor pursuits.

Relevant published literature in English was identified using searches of SCOPUS and Web of Knowledge. Formal literature searches of ‘title’, ‘abstract’, ‘keywords’ and ‘topic’ search fields were undertaken using the ‘(cow* OR bovine) AND (attack OR injury)’ search term, to May 2013 inclusive (figure 1).

### Media reports

Media reports from the UK newspapers describing cattle attacks were identified by searching Lexis Library (1993 to May 2013 inclusive) using the search phrase ‘(cow* OR bovine) AND (attack OR injury) AND (walker* OR public)’.

### Public guidelines

Current publically available internet guidelines describing best practice for walking among cattle were identified by searching Google, timespan unrestricted, UK pages only. The prepiloted search terms included: guidelines for walking through fields with cows; walking through fields with cows; cattle in fields crossed by public access; walking a dog through fields with cows; dog walking in cow fields.

## Results

### Published literature

Eight published papers reported outcomes and/or risk factors for cattle attacks (figure 1). Of these, six studies detailed injuries caused by cattle/bulls via hospital admittance.^7–12^ Two studies described bovine behaviour such as maternal defensive aggression/vigilance as a fear response to presence of a person/dog or movement to unfamiliar locations.^13^ ^14^

### Injury or fatality records

Kicking, crushing, head butting and trampling were the main causes of hospital-recorded injury, with fractures/contusions the most common consequences. Most injuries were associated with occupations involving direct cattle contact as opposed to public walkers, for example, dairy and beef farmers, vets and abattoir workers.^7^ ^8^ Fractures were the most common injury, kicking the most common mechanism, and feeding the most common situation.^8^ Injuries from bulls were more common than from cows.^9^ Specific bull-only incidents were also reported.^10^ ^11^ US data identified 287 bull cases; 261 were attacks on people, of which 149 were fatal.^11^ The injuries related to being charged or trampled, or accidentally stepped on^,^ by Holstein dairy and Angus beef dominant breeds.^11^

### Maternal behaviour

A study of maternal defensive aggression as a result of calves, a heritable trait, suggested that humans are perceived as a threat to calves, causing protective behaviour in cows.^13^ Extensification of pasture-based systems, decreased routine contact between handlers and non-lactating cows, an increase in calf contact post partum (to comply with EU ear-tagging regulations) and an increase, by the public, in recreational use of agricultural land were suggested as factors intensifying injury risk, with authors concluding an increasing trend in cattle maternal defensiveness and associated risk to handlers and public safety.^13^

### Fear of dogs

A study of vigilance to measure fear in dairy cattle concluded that dogs were even more threatening than unfamiliar humans, resembling potential predators, and cattle were exceptionally vigilant in novel locations.^14^

### Media

One thousand records were identified, 100 of which were relevant. Of these, 11 were duplicates, leaving 89 records (see online supplementary data).

**Figure 2:**
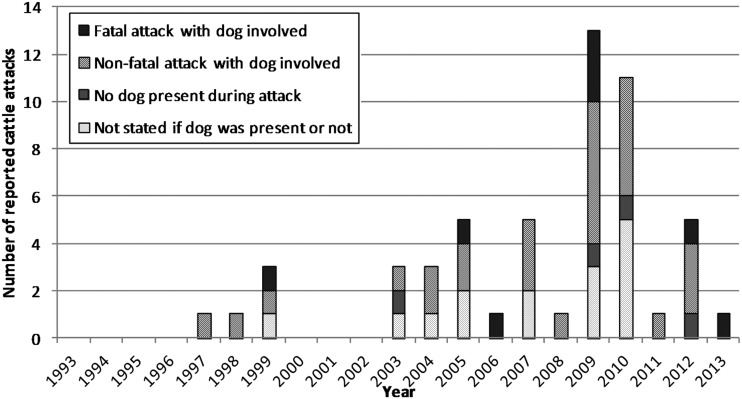
The number of cattle attacks on members of the public out walking in livestock areas reported in the media between 1 January 1993 and 31 May 2013, characterised by whether attacks were fatal and involved a dog.

From January 1993 to May 2013, 54 separate cattle attacks were reported involving the public walking in livestock areas (figure 2): attacks peaked at 13 in 2009, of which nine occurred in May and June. Thirteen (24.1%) attacks resulted in walker fatality, with a peak of four in 2009. Reported walker injuries included fractures to arms, ribs, wrist, scapula, clavicle, legs, lacerations, punctured lung, bruising, black eyes, joint dislocation, nerve damage and unconsciousness.

Most (72%) media reports definitively stated the presence/absence of dogs during an attack: 35 (64.8%) involved one dog, and there was no dog in four (7.4%). Reports also described five cattle categories: herd/multiple cattle (n=26, 48.2%); single cow (n=12, 22.2%); cows and calves (n=11, 20.4%); heifer (n=4, 7.4%); and bull (n=1, 1.9%).

Of the attacks, four were reported in more than five newspapers: one described an attack on David Blunkett, the former Home Secretary, who stumbled and broke a rib while releasing his guide-dog's lead; one described an attack on a veterinarian who was trampled and killed while protecting her dogs, who were thought to have sparked the attack; one described an attack on brothers, one of whom died and the other suffered a punctured lung while walking dogs; and one described an attack on a walker who required emergency neurosurgery. She had strayed from an obstructed public footpath to follow a diversion while dog walking; she picked up the dog after it startled cattle.

Media reports suggest factors influencing the likelihood or severity of attack including: the context as well as the behaviour of the people, dogs and cattle (table 1). Descriptive investigation of the data also suggests that female members of the public may be more likely to protect their dogs, while males let them go (see online supplementary data).

**Table 1:** Potential factors identified from media reports of cattle attacks on people out walking in livestock areas (1 January 1993 to 31 May 2013) which may have influenced the likelihood or severity of an attack

Context	Potential factors identified
Circumstances	Location If public footpath was followed Gender of the walker Age of the walker Walking alone or with others Walking with one or more dogs Dog on or off a lead Dog antagonised the cattle Action of the walkers towards the cattle Cattle type: herd/cattle; cows and calves; cow; heifer; bull
Behaviour of the walker (and dog)	Strayed from the right of way Running/walking/cycling Walked between a mother and calf Fell over Dog possibly sparked the attack Dog ran into herd Owner tried to rescue the dog Owner picked up the dog to protect it Owner let go of the dog's lead Owner put dog on a lead to walk around the cattle
Behaviour of the cattle	Attacked Trampled Charged Protecting calf Tossed person into the air Scared of, interested in or ignored the dog Kicked Stamped Knocked down Crushing Butting Gore Surrounded Startled Irate Running towards walkers Unknown

Sensational language is used throughout reports, with phrases such as ‘Cow attacks: “It looked like they wanted to kill him;”’^15^ such language depicts cattle as being dangerous and highly likely to attack at any point, while the walker is portrayed as blameless.

### Guidelines

Twenty-four separate web pages specifying countryside guidelines were identified, including not hanging onto dogs if threatened by cattle, releasing them; and avoiding getting between cows and calves (table 2). The content of the guidelines varied widely, however guidelines on best practice when dog walking stipulated in ‘The Countryside Code’^16^ were often reproduced. The Health and Safety Executive (HSE) also provides advice for those grazing cattle on public access fields, recognising the main risk factors: that walkers often have dogs; and when stressed by the weather, illness, unusual disturbance or when maternal instincts are aroused, cattle can become aggressive.^17^

**Table 2:** The most commonly occurring guidelines and advice obtained when searching for information using the ‘Google’ search engine, which describes best practice when walking through fields of cattle

Guidelines	Number of mentions	Sources
Leave gates and property as you find them	3	Countryside Code Natural England The Ramblers
Follow paths unless wider access is available such as ‘Open Access’ land	3	Countryside Code Natural England The Ramblers
Do not be afraid of cattle but be mindful they are protective of their young	1	NFU Cymru
Take a walking stick with you	2	NFU Cymru livefortheoutdoors.com
Be bold and walk straight through the cattle if the animals move towards you	1	NFU Cymru
Avoid getting between cows and their calves. Be prepared for cattle to react to your presence, especially if you have a dog with you	1	NFU and Ramblers
Move quickly and quietly, and if possible walk around the herd	1	NFU and Ramblers
Do not put yourself at risk. Find another way round the cattle and rejoin the footpath as soon as possible	1	NFU and Ramblers
Do not panic or run. Most cattle will stop before they reach you. If they follow, just walk on quietly	1	NFU and Ramblers
If you feel threatened, just carry on as normal, do not run, move to the edge of the field and if possible find another way round the field, returning to the original path as soon as is possible	1	go4awalk.com
Try to keep quiet and move away calmly and out of the field as soon as possible. Try not to surprise the cows	1	go4awalk.com
If cows get too close, turn quietly to face them with arms outstretched	1	go4awalk.com
Walk around cattle, rather than through them	1	livefortheoutdoors.com
If jostled by cows, turn to face them, wave your arms to make yourself look as big as possible and shout firmly	1	livefortheoutdoors.com
Apparently bulls can be controlled by twisting the ring in their nose	2	livefortheoutdoors.com walkingworld.com
If you want cattle to go away, wave your arms around and shout at them	1	walkingworld.com
Bulls: give it a reasonably wide berth and walk as quietly round it as you can (try not to run). If you have a close encounter with one you can (in theory) use the ring in its nose to control it, grab the ring and twist it	1	walkingworld.com
Keep dogs under effective control: On a lead, or in sight at all times, be aware of what it is doing and be confident it will return to you promptly on command. Ensure it does not stray off the path or area where you have a right of access.	5	Countryside Code Natural England NFU and Ramblers go4awalk.com livefortheoutdoors.com
If cattle chase you and your dog, it is safer to let your dog off the lead—do not risk getting hurt by trying to protect it	6	Countryside Code NFU Cymru NFU and Ramblers The Ramblers livefortheoutdoors.com walkingworld.com
Take extra care when walking dogs around livestock (especially young farm animals) Be especially vigilant if you find yourself with your dog in a field with both cows and calves	3	The Ramblers go4awalk.com walkingworld.com

Following three cattle-related deaths in 2009, the National Farmers Union provided new signage reminding walkers using public footpaths to keep dogs on leads, but release if chased or threatened by cattle.^18^ In October 2005, the Countryside and Rights of Way Act 2000 (CROW) was implemented across England^19^ giving the public freedom to roam without staying on paths; restrictions include keeping dogs on leads near livestock and landowners/tenants excluding or restricting access in certain circumstances.

## Discussion

Walking with dogs and cows with calves were key features of this study; both recognised as risk factors in the published literature, newspaper articles and web pages. Often dogs drew cattle's attention, and owners picked up dogs rather than releasing them.

Further, the enormity of the issue of cattle-related injury is unknown, although hospital records describe injuries mainly in animal husbandry and related occupations. This study confirms that walker injury from cattle is a public health risk worth future attention. There is no official documentation system, and it is likely that attacks are under-reported. Between April 1996 and March 2006, the HSE investigated 46 incidents,^20^ whereas the media review reported in this study identified 17 attacks, and at least one cattle attack requiring hospitalisation was not reported in newspapers nor investigated by HSE (Westgarth, personal communication). The lack of public concern about this risk is surprising considering the political and media concern over comparable animal-related injuries such as bites from dogs; however, cattle are not regarded as ‘members of the family’.

In conclusion, numerator information from cattle attacks gaining media attention are available but likely underestimate the true extent and no denominator information is available describing the number of countryside dog walkers; both are necessary to calculate the incidence of attacks and aid examination of risk factors. More research is required, in particular considering changes to countryside access and the promotion of physical activity for health. One possibility is the creation of a highly promoted central database, to encourage self-reporting of cattle attacks. Further, a generic approved guidance document in leaflet and web form would facilitate better management of this problem.

**What is already known on the subject**

- Many members of the public walk or participate in other outdoor pursuits in the UK countryside and they often have dogs with them.
- There are anecdotal and hospital reports of cattle causing injury or death to walkers.

**What this study adds**

- Injuries from cattle are a significant and under-reported public health risk.
- Walkers with dogs are at particular risk.
- Guidelines of how to behave around cattle and avoid injury vary, in particular concerning control of dogs.

## Supplementary Material

Web supplement
