# Supplementary material for: Are cattle dangerous to walkers? A scoping review
Source: Inj Prev. 2016 Jan 12;22(6):437–41. doi: 10.1136/injuryprev-2015-041784 (PMC5256230; doi:10.1136/injuryprev-2015-041784)
Supplement: Web supplement [file injuryprev-2015-041784-s1.pdf]

Key: m – male; f - female

Attack number	Number of papers reporting the attack	Attack date	Location	Public footpath	Walkers details	Category of cattle, as described by the media	Actions of the cattle, as described by the media	Presence of a dog and actions of the owner	Result of the attack
1	5	Nov-10	Nottinghamshire, Stanford on Soar	yes	2: m + f, not residents of the area	bull	Charged, attacked. The bull was later euthanised	N/A	m killed, f critically injured
2	13	Jun-09	Derbyshire, Peak District	N/A	2: m 62, son 26 (David Blunkett, former Home Secretary)	cow	charging, trample, attack, irate	Guide dog, black Labrador-cross, female, put on lead to walk around cattle. Owner tried to release dog but stumbled	broken rib, bruising
3	1	Oct-04	Derbyshire	N/A	f	cows	knocked down, kicked, stamped	N/A	punctured lung, broken ribs, severe bruising to face
4	1	Jul-11	Cornwall, St Dennis	N/A	4: f, m, 2 children	40 dairy cows	running towards the family	Jack Russell, male	Escaped by diving into a bush
5	1	Oct-05	not stated	N/A	3: m + 2 children, cycling	cow	gore the man	N/A	broken ribs, punctured lung, 3 days in intensive care
6	3	May-09	Beaminster	N/A	4: m, f 60, 2 friends	40 cows + calves	surrounded, butted, trampled, charged	2 Labradors, on short leads	broken ribs, stitches to mouth
7	2	Jun-12	County Durham, Romaldkirk	yes	2: m 46, f, holidaymakers	cows	attacked, trampled, reasons unknown	not thought to have a dog	m: serious injuries to head, back, arms, chest, abdomen. f: unhurt
8	2	Oct-10	North Yorkshire,	N/A	3: f 50s + m	cows	knocked to the	2 dogs	f: broken arm +

			Redmire		f 48<br><br>separate incidents on same day		ground, trampled	yes	fractured skull, m: minor cuts and bruises.<br>f broken ribs + collapsed lung
9	12	Jun-09	Yorkshire Dales, Pennine Way	N/A	f 49, vet	herd	trapped against wall, trampled	2 Spaniels and a Collie possibly sparked the attack. Owner tried to protect dogs	killed
10	4	Jul-09	Cardiff	no public right of way	f 63	herd, cows	trampled	Dog ran into herd. Owner went amongst the herd to put dog back on lead	killed
11	1	Jul-99	Derbyshire, Peak District	N/A	m 66	3 Charolais cows + calves	charged, attacked, protecting their calves	Golden Retriever, on lead, then let off when cows started charging	killed
12	1	Aug-09	Somerset, Burtle	N/A	m 75, farmer	herd, farmers own	scared by fire engine, trampled	no	killed
13	1	Jun-07	Hereford, Clehonger	yes	m, holidaymaker	herd of cows + calves	attacked: cow was standing on man's chest when he was found	dog	dislocated right knee, fractured left wrist, chest injuries
14	1	Jun-06	Pembrokeshire	yes	f	herd	knocked over, trampled	dog	killed
15	9	May-03	Cumbria, Greystoke	strayed from right of way: footpath blocked	f 45	20 Simmental-cross cows + calves	attacked, tossed in air, scared by dog	Jack Russell, on an extendable lead, startled the cows. Owner picked dog up to protect it	emergency neurosurgery for a head injury, fractured arm + ribs, knee injury
16	2	May-09	Bristol, Wapley Wood	yes	m 42	herd: > 20 cows + calves	trampled	Jack Russell-Spaniel cross, m, on lead. Owner let go of lead during attack	broken ribs, severe bruising, black eyes, large lump on head
17	1	Jul-09	Bath	N/A	m	cow	trampled	N/A	spinal injuries

18	3	Jul-99	Lancashire, Whalley	yes	m 41, postman	heifer + calf	charged, protecting her calf	N/A	severe spinal injuries
19	2	Aug-10	East Sussex, Burwash	yes	f 42	herd, 6 cows	trampled	Schnauzer, on lead. Owner let go of lead	broken scapular, displaced clavicle, 3 broken ribs, nerve damage, hoof mark on knee
20	1	Aug-07	East Sussex, South Downs	N/A	m, Inspector	50 beef cattle	butted, stamped	N/A	4 broken ribs, punctured lung
21	2	Jul-10	Gloucestershire, Forest of Dean	N/A	m 55, holidaymaker	cattle	trampled	Airedale Terrier	serious head injuries
22	1	Jun-12	West Yorkshire, Norwood Green	yes	2: m 49 + son 9	30 to 40 cows	N/A	Dog, on lead, escaped	m unconscious but ok, son escaped
23	1	Sep-10	London, Totteridge	yes	10: pensioners	Friesian cow	N/A	N/A	1 f: broken leg, 2 with severe bruising
24	2	Oct-12	Wiltshire, Chippenham	N/A	f 68	herd	trampled	dog: treated for injuries	killed (cardiac arrest)
25	2	Oct-12	Wiltshire, Cherhill White Horse	yes	f 57, nurse	cow	trampled	2: Rottweiler and Rottweiler-German Shepherd cross, off the lead	extensive bruising, fractured ribs + fractured shoulder blade
26	2	Jun-97	Windermere, Dales Way	yes	m, running	herd of cows	attacked	dog	face sewn back together, lungs drained, 7 broken ribs
27	1	Sep-04	Derbyshire, Little Longstone	yes	f	herd: 6 cows + calves	trampled	dog	punctured lung, 2 broken ribs, bruising to face
28	2	Oct-07	Brighton	yes	m 50	50 + calves	interested in dog, trampled	Golden Retriever, on lead. Owner fell and	life threatening, punctured lung,

								let go of lead. Dog ran away, distracting the cows	broken rib severed an artery
29	1	Jun-09	Suffolk, South Elmham	N/A	f 45	herd of cows	trampled	2. Owner tried to bring dogs under control	killed
30	1	Apr-98	Oxfordshire, Bloxham	N/A	f 40s	herd of cows	trampled	Border Terrier. Owner tried to rescue dog	broken collar bone, punctured lung, broke all 24 ribs
31	1	Oct-07	Bedfordshire, Leighton Buzzard	N/A	m 73, retired doctor	cattle	N/A	N/A	killed
32	2	Sep-10	Cambridgeshire, St Neots Common	N/A	m 59	cows	trampled, attacked	dog	serious chest injuries
33	2	Jul-04	Cumbria, Askam-in-Furness	N/A	f 82	cows + new-born calves	attacked to protect calves from dog	Border Collie	bruising, broken ribs
34	2	Jun-08	Cheadle, Kingsley	yes	2: m 60 + friend	cow	N/A	Spaniel	m: head injury, multiple rib fractures, bruising to back and abdomen
35	1	Jun-09	Scotland	N/A	f	cow	charged	N/A	broken ribs
36	1	May-09	Isle of Man, Colby	yes	f 52	cow	startled	dogs	6 broken ribs, punctured lung
37	1	Jul-10	France, Pyrenees	N/A	f 37	cows: several	trampled	N/A	unconscious
38	1	Jul-10	France, Pyrenees	N/A	m 50, climbing	cow	charged	N/A	head injuries, cuts to arms
39	1	Jun-09	Sussex Downs	N/A	m, police inspector	30 cows	crushed	dog	crushed
40	1	Oct-05	Oxford, Godstow Lock	N/A	2: m 72 + m	brown heifer, among a group of 10 cows + 2 calves	N/A	dog, on lead	used a walking stick and branch to fend off the cow
41	2	Aug-05	Oxford, Godstow	N/A	3: m 41 +	French Saler	N/A	N/A	punctured lung,

			Lock		children; m 14, f 13; cycling	heifer, among a group of 10 cows + 2 calves			broken ribs
42	1	Sep-05	Pembrokeshire	N/A	f 55	40 suckler cows + calves	trampling, crushing, butting	dog	found dead, suffered injuries consistent with trampling, crushing and butting
43	1	Oct-12	Shropshire, Oswestry	N/A	m 46	herd	charged, trampled	dog	face, chest and leg injuries
44	1	Aug-03	Scotland, Kyle of Lochalsh	N/A	m	cattle + calves	N/A	no	died
45	1	Apr-05	Derbyshire, Baslow Edge	N/A	m 58	highland cow	N/A	Dog. Owner walked between mother and calf	6 broken ribs
46	1	Oct-03	Wolvercote, Port Meadow	N/A	f 60	cow	N/A	N/A	2 black eyes, bruising
47	1	Jun-10	Derbyshire, Middleton - by - Youlgreave	N/A	m 78	herd	N/A	N/A	died
48	1	May-09	East Sussex, Guestling	yes	m 34	cows	N/A	Bulldog; catapulted into the air by a cow, died as a result.	broken ribs, bruising
49	1	Nov-99	Dartmoor	N/A	f 31	Black Galloway cow	N/A	2 miniature Dachshunds, on leads tied to owners waist, ignored by cow	leg, head and internal injuries
50	1	Aug-10	East Sussex, Colemans Hatch	N/A	f	Charolais	N/A	no	ankle injury
51	1	Sep-10	Yorkshire, Dales Way	yes	2: m 66 + f	heifer	ignored the dog	3 dogs, let off the lead	bruising, cut to face, suspected cracked rib
52	1	Oct-09	Yorkshire, Dales Way	N/A	f	cow	N/A	N/A	N/A

53	1	May-07	Devon, Culmstock	yes	f 62	cows	trampled	dog	serious chest injuries
54	10	May-13	Wiltshire, Elbow Field	N/A	2: m 65 + m 72	24 adult crossbreed cattle: mixture of Friesian and Limousin	trampled	dogs	m 65: died<br>m 72: collapsed lung, ‘stable’ condition
